# A Comprehensive Analysis of Organic Food: Evaluating Nutritional Value and Impact on Human Health

**DOI:** 10.3390/foods13020208

**Published:** 2024-01-09

**Authors:** Azizur Rahman, Parnian Baharlouei, Eleanor Hui Yan Koh, Diana Gabby Pirvu, Rameesha Rehmani, Mateo Arcos, Simron Puri

**Affiliations:** 1 Centre for Climate Change Research, University of Toronto, ONRamp at UTE, Toronto, ON M5G 1L5, Canada; parnian.baharlouei@mail.utoronto.ca (P.B.); eleanor.koh@mail.utoronto.ca (E.H.Y.K.); gabrielapirvu@hotmail.com (D.G.P.); rameesha.rehmani@mail.utoronto.ca (R.R.); mateo.arcos@mail.utoronto.ca (M.A.); simron@climatechangeresearch.ca (S.P.); 2 A.R. Environmental Solutions, ICUBE-University of Toronto, Mississauga, ON L5L 1C6, Canada; 3 Physiology and Human Biology, University of Toronto, Toronto, ON M5S 1A8, Canada

**Keywords:** organic foods, food safety, obesity, cancer, biodiversity, climate change, organic farming

## Abstract

In recent years, organic agriculture has gained more popularity, yet its approach to food production and its potential impact on consumers’ health and various environmental aspects remain to be fully discovered. The goal of organic farming practices is to maintain soil health, sustain ecological systems, maintain fairness in its relationship with the environment and protect the environment in its entirety. Various health benefits have been associated with higher consumption of organic foods. This review identified some of these health benefits, including a reduction in obesity and body mass index (BMI), improvements in blood nutrient composition as well as reductions in maternal obesity and pregnancy-associated preeclampsia risks. Furthermore, organic food consumption can reduce the development of non-Hodgkin lymphoma (NHL) and colorectal cancers. Upon reviewing the existing literature regarding the nutritional value of organic foods, it was found that organic food contained higher levels of iron, magnesium and vitamin C. However, the evidence available to draw definitive causations remains limited due to study biases, short study durations and confounding variables; thus, it cannot be concluded that the organic diet provides any related health benefits. In this review, we provided essential insights and statistical analysis from the evidence available and consider study limitations to evaluate the potential of organic food consumption in positively impacting human health.

## 1. Introduction

Organic farming is designed to mitigate environmental pollution and prioritize animal welfare through protective management strategies that prevent exposure to harmful pesticides, industrial solvents and synthetic chemicals [1,2]. However, this system of management goes beyond avoiding the use of synthetic inputs by basing its practices on four principles: health, ecology, fairness and care [3,4]. The principle of health ensures that organic agriculture should sustain and strengthen the health of the soil, plants, animals, humans and the earth as a whole [3]. The principle of ecology focuses on living ecological systems and how organic agriculture should work with, sustain and emulate these systems [3,5]. The principle of fairness underscores the importance of relationships ensuring fairness in the common environment and life opportunities [3]. Finally, the principle of care advocates for safe and responsible agricultural management to protect current and future generations and the environment [3]. To adhere to these principles, organic farming employs practices such as crop rotation, intercropping, polyculture, covering crops, seeding timing and mulching [3]. Notably, the increasing awareness and demand for organic food in recent years are attributed to its perceived health benefits and positive impact on environmental biodiversity [6,7,8,9,10,11,12,13,14,15].

The primary motivation for purchasing organic food is its perceived health benefits, followed by considerations for ecosystems and the environment [11,12,13,14,15,16,17]. Consequently, the global organic food market has experienced rapid growth, with an estimated 10% increase since 2000 [18]. Since then, the organic food production market was valued at CAD 7 billion in 2020 and organic packaged food sales are projected to reach USD 1.6 billion by 2025 [19]. Considering the rapidly growing demand for healthy, environmentally conscious foods, it is important to explain how public perception of the organic diet has influenced its surge in popularity.

Generally, several reports have uncovered that consumers who strictly follow an organic diet do so for one of several reasons: perceived health benefits, concern for the environment and the inherent value of buying local [11,15,16,17,19,20]. Health-conscious consumers are more likely to avoid mainstream products containing pesticides, hormones, and other additives, instead opting for organic alternatives that are marketed as natural and chemical-free [15,16]. Correspondingly, Rana and Paul discovered that Canadians placed a lot of value on the certification and labeling of the organic packaged goods they were buying [21]. Comparatively, concerns about accessibility, safety, and price were predominant in Slovenia, Portugal and China [21]. Some Canadian organic consumers even had preferences for particular organic certificates and commonly sought information about the product’s origins and the production methods used [21]. Thus, consumer trust in the product they are purchasing heavily influences their decision to buy organic [11,12,13,15,19,20,21].

Conversely, the higher cost, lack of widespread availability and lack of perceived value were all reported to be factors that deterred consumers from purchasing organic foods [15,19,20,21]. Previous studies have discovered that organic foods are on average 10% to 40% more expensive than conventionally produced foods [22]. Further on, a 2021 online survey of up to 187,000 Canadians demonstrated that 18% of respondents believed organic foods were no different from mainstream products [19], possibly because the evidence surrounding their health benefits remains ambiguous.

**Figure 1:**
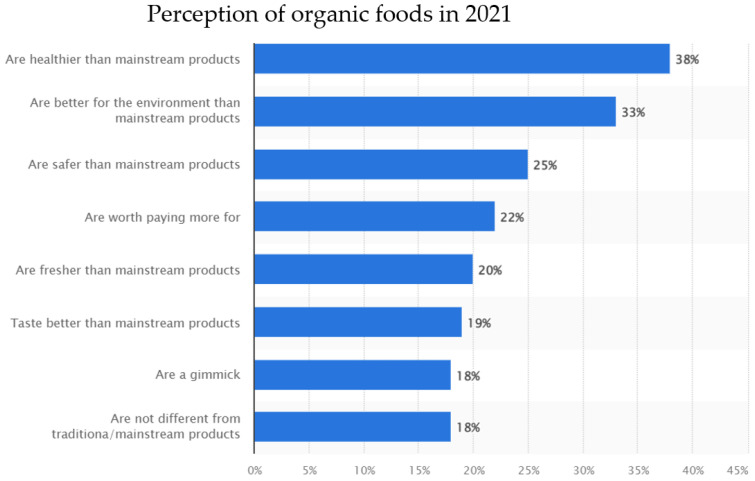
Breakdown of opinions on organic food in Canada. Data collected from a 2021 online survey of up to 187,000 Canadians over 18 years of age [19].

Considering affordability and perceived value majorly influence purchasing decisions, higher income levels often correlate with an increased likelihood of purchasing organic foods [13,21,22]. In addition, higher levels of education are associated with greater awareness of health and environmental concerns related to food choices [13]. Educated consumers may be more informed about the benefits of organic farming practices and choose organic products accordingly. A recent study investigating the organic purchasing intentions of Bangladeshi consumers uncovered a significant positive correlation between the level of education and the intention to purchase sustainable organic food. Specifically, the study found a 3.27-fold increase in organic food purchasing among consumers with higher levels of education [13]. Other socio-economic factors that may influence organic purchasing decisions include age and gender, cultural dietary habits and health and wellness trends in the market [11,12,13,14,15,16,20,21]. For example, the same study demonstrated that Hungarians and Swiss people over the age of fifty are more price sensitive [13]. In addition, some cultural or ethnic groups may have traditions or preferences for specific types of organic produce or traditional farming methods, such that individuals with specific health concerns or dietary preferences may opt for organic options. Finally, growing health consciousness and a focus on wellness can drive the demand for organic foods perceived as healthier and free from synthetic chemicals [13,14,15]. Figure 1, taken from Statista 2021 [19], further breaks down the surveyed consumers’ attitudes toward organic products and provides further insights into how organic food is perceived in Canada.

This review article aims to elucidate key impacts of organic agriculture on human health and provide insights into current market trends. Given that food safety is a pivotal determinant influencing consumer choices [23], our investigation focuses on exploring the ramifications of embracing organic farming practices to ascertain whether such practices can indeed yield favorable health outcomes for organic consumers. Our analysis encompasses an examination of findings from various studies conducted over the past 25 years, combining original research and cohort studies sourced from public literature to present a comprehensive overview of the potential impact of organic food consumption on human health.

## 2. Organic versus Conventional Food 

**Figure 2:**
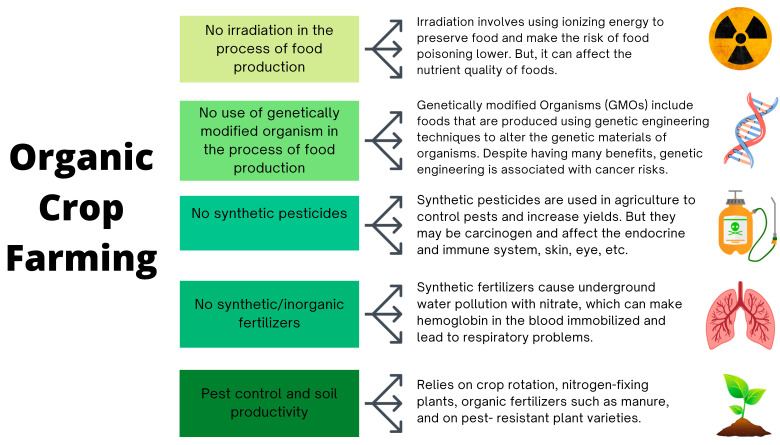
Organic crop farming at a glance [29,30,31,32,33].

The production of organic food requires special considerations (Figure 2). Generally, organic farming is solely grounded in biological and ecological processes that mitigate the environmental impact of agricultural practices while preserving the natural qualities of food [10,24]. In this holistic approach, pest and disease control are achieved naturally, eliminating the need for synthetic chemicals utilized in conventional farming [24,25]. Additionally, organic food must not be sourced from genetically modified organisms (GMOs) [24,26]. Organic farming also relies on mechanical weeding as an alternative to traditional herbicide input, potentially leading to increased weed cover that benefits various organisms by promoting biodiversity [26]. Core principles of organic agriculture, such as the use of green manure, crop diversification, and small fields, further contribute to the production system’s sustainability [26]. Following these principles, organic farming is believed to enhance soil fertility and foster biodiversity. Studies indicate that local species richness and abundance can increase by approximately 34% and 50%, respectively, across various crops worldwide compared to conventional farming practices [26]. Thus, there has been a recent upsurge in both the production and purchasing of organic goods, driven by a heightened demand for natural products that undergo minimal processing and abstain from synthetic and artificial fertilizers or pesticides in their production processes [27,28].

Unlike conventional farming, organic farming does not use genetic engineering or synthetic pesticides in the food production process, allowing for an assessment of their health effects. The use of genetic engineering and GMOs can pose various health risks, such as allergic reactions and unexpected interlinks between genes due to gene additions and modifications [31]. Moreover, pesticides utilized in agriculture can accumulate in soil and water, quickly entering the food chain and impacting human health [32]. These health effects span from allergic reactions to lung damage, causing breathing difficulties, nervous system problems, birth defects, and the risk of chronic diseases such as cancer [32]. For instance, the organochlorine insecticide (OCI) dichlorodiphenyltrichloroethane (DDT) functions by opening sodium channels in the human nervous system, leading to increased firing of action potentials that can result in spasms and, in severe cases, death [34,35]. Conversely, carbamate insecticides inhibit the acetylcholinesterase enzyme, interfering with cell replication and differentiation, proper synapse signaling, and other neurotoxic effects [34,36]. Furthermore, the growth regulator herbicide 2,4-D, used to eliminate weeds, has been linked to severe eye irritations and fertility problems in men [34]. Studies have also associated anilide/aniline herbicides with risks of colon and rectal cancer [34]. Glyphosate, a common ingredient in pesticides found in GM crops, has also been linked to cancer risks, especially non-Hodgkin lymphoma (NHL) [31]. Glyphosate was first used as a broad-spectrum pesticide in 1974 [37]. As genetically engineered glyphosate-tolerant crops were introduced, glyphosate quickly spread worldwide and has now become the most widely used pesticide in agricultural and residential sectors [37]. However, glyphosate is an organophosphorus compound which interferes with aromatic amino acid synthesis through a mechanism unique to plants [37]. Thus, concerns have arisen about glyphosate’s potential genotoxicity through the induction of oxidative stress for human cells in vitro and in animal experiments [37]. A 2021 review on the health effects of glyphosate stated a clear association between glyphosate exposure and a wide range of human diseases, including gut microbiota dysbiosis, kidney and liver damage and neurological conditions such as Alzheimer’s [38]. 

It is important to note that a majority of these experiments tested much higher doses than those permitted for agricultural use. Notably, esteemed institutions such as the Food and Agriculture Organization (FAO), the European Chemicals Agency (ECHA) and the European Food Safety Authority (EFSA) have affirmed glyphosate’s status as non-toxic and non-carcinogenic to human target organs as of 2022 [38]. In rabbit studies conducted by the EFSA, an acceptable daily intake (ADI) of 0.5 mg/kg of body weight per day was defined, while the FAO and WHO established an acute toxicity measure (LD50) of 5600 mg/kg of body weight for the oral pathway and over 2000 mg/kg of body weight for the dermal pathway [38]. Consequently, the commercialization of glyphosate-based herbicides (GBHs) is subject to stringent regulations, including the establishment of maximum residue limits (MRLs) for glyphosate residues in various food items. Despite these regulations, an EFSA multinational study identified glyphosate in 24 out of 186 honey samples, with 8 surpassing legal limits (Table 1) [38]. Approximately 30% of honey samples in the USA contained glyphosate residues, with over half exceeding MRLs—including one sample that was seven times higher than the allowed limit (Table 1) [38]. Further on, studies in Canada and Switzerland found detectable levels of glyphosate in nearly all samples, although concentrations remained below the MRL of 50 μg/kg [38]. 

Considering the MRL for pesticides is typically determined through testing individual pesticides on rats for a relatively brief duration, there is a substantial lack of knowledge regarding the consequences of consuming potentially hundreds of different pesticides over one’s lifetime. The intricate interplay of these various pesticides remains largely unknown. Thus, further research is needed to uncover the cumulative long-term health effects of glyphosate and other pesticide residues, as many studies reveal a variety of toxic effects [38]. As a result, pesticide use may adversely affect human cells through mechanisms still unclear, and it may be possible to minimize these health risks by re-orienting agricultural practices toward more organic approaches. 

### 2.1. Nutritional Benefits

The nutrient and mineral content of crops is affected by various agronomic factors including fertilization type, crop rotation designs and crop protection protocols [24,39]. For example, the addition of organic matter to soil helps provide food for beneficial plant microorganisms, and in return, these stimulated microorganisms produce valuable compounds (including citrate and lactate) that make soil minerals more available to organic plant roots [39]. In addition, organic farming allows for the slow release of soil minerals over time, causing essential nutrients to become available when needed, whereas chemical fertilizers quickly dissolve in irrigation water and deliver excess quantities of nutrients to crops, often past what is needed [39]. Thus, agronomic differences in organic versus conventional farming systems may impact the quantity and quality of beneficial compounds that can be obtained from each crop type [24,39]. However, studies comparing the nutrient content between organic and conventional crops have revealed inconsistent results [24]. Further on, many of these studies lack the necessary control factors to validate the results, such as failing to consider the different environmental and growing conditions that affect crop quality [24]. In 2012, Smith-Spangler et al. [29] reviewed the results of 223 studies examining the nutrient content of organic foods, including ascorbic acid, phosphorus, calcium, magnesium, iron and various vitamins. The findings showed that organic fruits, vegetables, and grains do not exhibit significantly higher nutrient levels compared with their non-organic counterparts. However, organic produce did show higher levels of phosphorus when compared with non-organic produce [29]. All in all, the evidence was not strong enough to suggest that organic foods are more nutritious than non-organic foods. However, further recent experiments [40,41,42,43] have demonstrated that some organic foods, such as corn grain, wheat flour, broccoli, tomato, black sesame and leafy vegetables, contain more minerals and vitamins, which are discussed below.

### 2.2. Mineral Content

The most essential minerals are calcium, magnesium, potassium, iron, zinc, copper, manganese, selenium and iodine [40]. Studies have shown that the content of these minerals in fruits, especially apples, does not differ significantly between organically grown and conventional methods [40,41]. Studies on organic vegetables, however, revealed higher levels of iron and magnesium compared to conventionally grown vegetables. Overall, Worthington revealed that the iron and magnesium content in organic crops was higher by 21% and 29%, respectively [39]. Moreover, a study by Yu et al. [42] demonstrated 20% higher magnesium content and 30% higher phosphorus and potassium contents in organic compared to conventionally grown summer corn. However, the study did not provide details on methodologies or sample sizes, thereby limiting the credibility of the reported data. They also found higher levels of zinc and iron in organic corn, but this increase was not significant [42]. These findings were further compounded by Rembialkowska [43], where the results of many experiments demonstrated a higher level of iron, phosphorous and magnesium content in organically grown compared to non-organically grown products. These results may be attributed to the effects of traditional potassium fertilizers used in conventional agriculture, which can decrease the amount of magnesium—and consequently, phosphorus—absorbed from soils [39]. Further on, organic fertilizers tend to increase the number of soil microorganisms that affect various components of plant nutrient acquisition and metabolism, which may play an essential role in making iron more bioavailable to plant roots [39]. Confounding factors, including variations in soil fertility, pH levels and the presence of specific minerals across different plots and geographical regions, can significantly influence the absorption and availability of nutrients for plants [39]. Consequently, any observed differences in the nutritional content of organic and conventional produce may be attributed to variations in soil conditions and cultivation practices rather than the farming methods alone. To mitigate these potential confounding variables, researchers must meticulously control and monitor soil conditions, cultivation practices and climatic variations in their study to ensure that the comparison between organic and conventional crops is not influenced by any disparities in these factors. 

### 2.3. Vitamin Content

Experiments on the various vitamin contents of different organic versus non-organic fruits and vegetables are limited. A 2010 review on the nutritional quality of organic food revealed higher vitamin C contents in organic potatoes, tomatoes, kale and celeriac as well as higher vitamin E content in organic olive oil [40]. Similarly, Worthington’s experiment revealed 27% higher vitamin C levels in organically grown lettuce, spinach, potatoes and cabbage [39]. On the other hand, some studies on beta-carotene (vitamin A precursor) have shown that the beta-carotene content of organic foods greatly depends on the type of fertilizer used, as nitrogen fertilizers have been shown to yield higher beta-carotene levels in carrots [40,41]. Other experiments have shown similar outcomes in conventional agriculture, such that increased fertilization changes the content of secondary plant metabolites [44]. For example, Mozafar [45] revealed that nitrogen fertilizer used in conventional fruits and vegetables could increase the amount of beta-carotene and reduce vitamin C levels. This phenomenon can be attributed to alterations in plant metabolism observed in response to the differences between organic and conventional fertilizers. For example, when exposed to a high influx of nitrogen, plants tend to increase protein production while diminishing carbohydrate production, ultimately leading to a reduction in vitamin C synthesis [39]. Consequently, the vitamin content in crops is significantly influenced by the specific agronomic factors associated with each farming system.

### 2.4. Other Compounds

Oxidation of phenolic compounds by the polyphenol oxidase (PPO) enzyme is part of the plant antioxidant defense mechanism (to repair injuries on their surface). Phenolic compounds act as a chemical barrier against invading pathogens. Intact antioxidant defense in plants has been shown to have important implications for human health, including playing an anticarcinogenic role [42]. Organic cultivation operations have been revealed to increase the polyphenol content of peaches and pears as compared with their conventional counterparts [9]. Moreover, increased activity of the PPO enzyme towards chlorogenic and caffeic acids (antioxidant agents) was observed to be notably higher in the organic samples of peaches and pears [9]. Overall, various studies on organic crops have observed between 18% and 69% increased antioxidant activity in these products [46]. Intake of antioxidants and phenolic compounds from food consumption is important because these compounds have been shown to effectively reduce the risk of chronic diseases, including some neurodegenerative and cardiovascular diseases and cancer [46]. 

Another organic compound that has increased in quantities within organic foods is salicylic acid. Salicylic acid is a metabolic component of aspirin and has a high anti-inflammation capacity [47], and its intake from dietary sources has beneficial health effects. Aspirin and its metabolites, including salicylic acid, can reduce the risk of cardiovascular diseases and reduce up to 40% of the risk of colorectal cancers [48]. Relevantly, organic practices have been shown to increase the salicylic acid content of vegetable soups in comparison to their conventional counterparts [47], as displayed in Table 2 [42,47].

Importantly, organic foods also demonstrated lower levels of toxic metabolites, such as cadmium and pesticide residues [49]. Cadmium is a heavy metal that is known to accumulate in the body and exert toxic effects on the kidneys and liver [49]. Importantly, eight meta-analyses conducted by Barański et al. revealed that organic crops contained on average 48% lower cadmium concentrations than conventional crops [49]. Further on, the frequency of detectable pesticide residues was four times lower in organic crops, whereas the frequency of phenolic (antioxidant) compounds was on average 20–40% and, in some cases, over 60% higher in organic crops [49]. The study analyzed a comprehensive dataset comprising 343 peer-reviewed publications, where notable discrepancies emerged across different crop types, crop species, and studies conducted in countries with different climates, soil types and agronomic backgrounds. Thus, potential limitations of these meta-analyses include variations in study methodologies and geographical locations that confound the observed results. However, by employing the GRADE (Grading of Recommendations, Assessments, Development, and Evaluation) assessment to gauge the strength of evidence for a standard weighted meta-analysis, the overall strength of evidence was deemed moderate or high for the majority of parameters where significant differences were identified (i.e., many phenolic compounds, cadmium and pesticide residues) [49].

Accordingly, a French BioNutriNet case-control study investigated the difference in urinary pesticide metabolite concentrations between 150 high-organic-food consumers and 150 low-organic-food consumers, matched for dietary patterns and other relevant traits [50]. Notably, the authors saw significant reductions of organophosphrous pesticides (OPs), diethyl-thiophosphates, dimethylthiophosphase, dialkylphosphates (DAPs) and free 3-phenoxybenzoic acid in the high-organic-consumer group, ranging from –17% to –55% reductions compared to the low-consumption group [50]. These differences were attributed to fertilization techniques, crop protection regimens, and other agronomic factors between growing practices. For example, organic farming systems avoid the use of fertilizers produced from industrial waste, which are often the most contaminated by toxic heavy metals [39]. 

Together, these results indicate that it may be possible to minimize dietary cadmium and pesticide intake levels by switching to an organic diet. However, there is no evidence to suggest that non-organic foods contain significant concentrations of pesticides or toxic metals that pose a risk to human health or that reduced exposure through high organic consumption is preventative for any specific health concern. Thus, several studies have demonstrated that the nutritional contents of select organic foods significantly differ compared to conventionally grown foods (Table 2 [42,47] and Table 3 [39]), although the associated health benefits of these differences are not well-established. 

## 3. Impact on Human Health

The findings from clinical experiments assessing the health impact of organic food on humans are relatively limited compared to other nutritional epidemiological studies. Many of these experiments are short term and may be confounded by variations in dietary patterns and lifestyles that profoundly affect human health [51]. Notably, observational studies often lack a comprehensive examination of the various health factors that may differ between organic and non-organic food consumers, such as lifestyle choices, physical activity levels and overall dietary patterns [50,51]. These factors may be a source of confounding that significantly influence the health outcomes observed, precipitating the need for further longitudinal intervention studies. Nevertheless, the compounds found in organic fruits and vegetables are generally believed to promote human health and longevity [51]. Consequently, individuals who consistently consume organic food often opt for more fruits and vegetables and less meat, potentially reducing the risk of mortality and chronic diseases [52,53,54,55,56,57]. Additionally, research indicates that those who regularly choose organic food are more likely to be female, have higher education and income levels and maintain a healthier lifestyle by smoking less and engaging in more physical activity [50,51,58,59]. As a result, the dietary compositions of organic and non-organic consumers may significantly differ. This section aims to present evidence from studies that have assessed the impact of organic food on human health outcomes, with consideration for the potential biases and limitations that can affect results.

## 4. Epidemiological Findings Related to Human Health

### 4.1. BMI and Obesity

**Figure 3:**
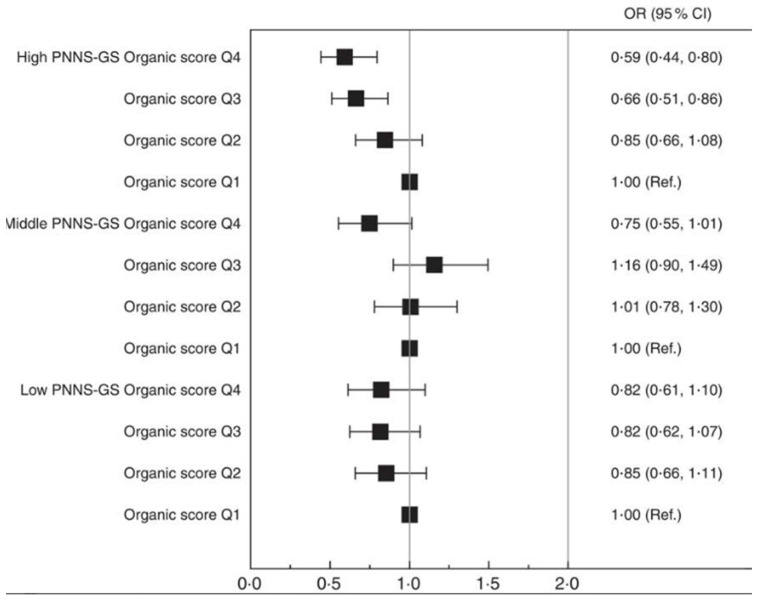
The prospective association between the organic score in quartiles (Q) and the risk of obesity, represented as % BMI change from the first quartile (Q1, baseline = 1.0). Organic scores are stratified according to diet nutritional quality and based on a low, middle, or high Programme National Nutrition Santé guidelines score (PNNS-GS). Values are OR and 95% CI, adjusted for age, sex, month and year of inclusion, delay in follow-up, occupation, marital status, education, monthly income per unit, dietary supplement use, modified Programme National Nutrition Santé guidelines score (mPNNS-GS), principal-component-analysis-extracted dietary patterns scores, energy intake, physical activity, tobacco status and history of chronic diseases. Ref. = referent values. Taken from Kesse-Guyot et al. (2017) [62].

Body mass index (BMI) is a weight-to-height index that divides an individual’s weight (kg) by their height (m^2^), providing a valuable indicator for determining obesity and overweight in adults [60]. The WHO defines obesity as a BMI equal to or greater than 30 in adults, while overweight is classified as a BMI equal to or greater than 25 in adults [61]. In a prospective cohort study conducted in 2017, the Nutri-Net Santé Cohort analyzed self-reported dietary and anthropometric data from 62,224 French participants to determine how organic food consumption affects obesity risk [62]. Participants were assigned an organic score based on their organic consumption frequency, and these scores were divided into four quartiles, with the first quartile (Q1) serving as a baseline for modelling BMI changes. Models were adjusted for several characteristics, including sex, income, energy intake and expenditure, history of disease and baseline use of dietary supplements. Upon assessing the association of the organic score with BMI change through ANCOVA, the researchers discovered a significantly positive association between high organic food consumption and a reduced risk of being overweight (OR = 0.77, 95% CI 0.68, 0.86, *p* < 0.0001) [62]. This association remained highly significant in a 3.1-year follow-up study that demonstrated a 37% reduced risk of obesity in the high organic consumption group [62]. Specifically, males who regularly consumed organic foods exhibited a 36% and 62% lower probability of being overweight and obese, respectively, while females who regularly consumed organic foods showed a 42% and 48% lower probability compared to non-consumers [62]. Overall, their results demonstrated a strong reduction in the risk of being overweight and obese among high-frequency organic food consumers, as depicted in Figure 3 [62]. In particular, this association was stronger in participants who reported consuming more nutritious diets, as assessed by the Programme National Nutrition Santé-guidelines score (PNNS-GS) (Figure 3). Observed associations remained significant even after accounting for selection bias by inverse probability weighting. However, it is essential to acknowledge the inherent challenges in designing and conducting observational studies. The reliance on self-reported dietary and anthropometric data introduces potential recall biases, raising concerns about the accuracy and reliability of the information. These challenges should be recognized and considered when interpreting the findings from such studies. 

Another cross-sectional study by Perez-Cueto et al. was conducted to compare food-related lifestyles (FRLs) between 2437 obese and non-obese respondents in five European countries (Belgium, Denmark, Germany, Greece and Poland) [63]. According to their experiment, obese participants scored lower on most dimensions of FRL related to food quality, particularly organic products, suggesting that eating more organic products reduces obesity risk.

Furthermore, a cross-sectional BioNutriNet project [64] in France comprised of 5855 participants, including children, adolescents, and adults, assessed the relationship between organic food consumption and obesity over a one-year period. Employing a three-stage stratified random sampling approach, data on food supplement usage, dietary patterns, physical activity, sedentary behaviors, health conditions, sociodemographic traits and height and body weight measurements were collected through structured face-to-face questionnaires. The results showed that in all age groups, higher consumption frequency of organic food was associated with lower BMI and obesity—however, the strength of this relationship was reported to be small [64]. An additional study examined the association between organic food consumption and obesity risk among 37,706 Sister study participants between 2003 and 2009 [65]. The participants in the age range of 35–74 reported eating organic food (including meat, dairy and produce) never, less than half of the time, about half of the time or more than half of the time in the past 12 months. The organic diet score (ODS) was calculated based on the frequency of organic food consumption, with a higher score indicating more frequent consumption. The researchers compared BMI at the time of enrollment and over a mean 8.3-year follow-up and found not only that women who ate organic foods had lower baseline BMI but also that eating less organic food was inversely related to weight gain [65]. 

Overall, these studies have demonstrated the association between organic food consumption and reduced risk of obesity. However, issues regarding the validity and accuracy of self-reported data come into question. Further on, these associations cannot prove causation, as BMI is heavily influenced by overall dietary quality and other healthy lifestyle habits that frequent organic consumers are typically more conscious of. The French BioNutriNet study, among others, made efforts to address various confounding variables, including socio-economic status, energy intake and expenditure, lifestyle factors, and inherent biases in observational research. To mitigate information bias, the study assessed the convergent validity of the organic food index and objectively measured height and weight. Additionally, a comprehensive survey design was employed to ensure the representativeness of the sample and minimize selection bias. However, future work is required to investigate the influence of residual confounding factors on the observed relationship between organic food consumption and BMI, given the well-established correlation between obesity and mental health issues such as depression or drug addiction [64]. Moreover, given that the questionnaire only covered a span of a year, it is essential to acknowledge that BMI and obesity status are influenced by a nutritional history extending beyond the previous year. Therefore, further longer-term longitudinal studies are imperative to yield crucial insights into our understanding of obesity risk and organic food consumption.

### 4.2. Blood Composition

Clinical studies have demonstrated that individuals who consume a high amount of organic food exhibit more favorable blood compositions compared to infrequent consumers. 

Notably, the Nutri-Net Santé nested case-control study also revealed higher nutritional content in the fasting blood plasma samples of frequent organic food consumers [50]. Plasma levels of magnesium, fat-soluble micronutrients (a-carotene, b-carotene, lutein and zeaxanthin), fatty acids (linoleic, palmitoleic, g-linoleic and docosapentaenoic acids) and some fatty acid desaturase indexes were found in greater concentrations in frequent organic food consumers [50]. In contrast, no measurable differences were detected for other carotenoids such as lycopene and β-cryptoxanthin, minerals iron and copper or vitamins A and E [50]. 

**Figure 4:**
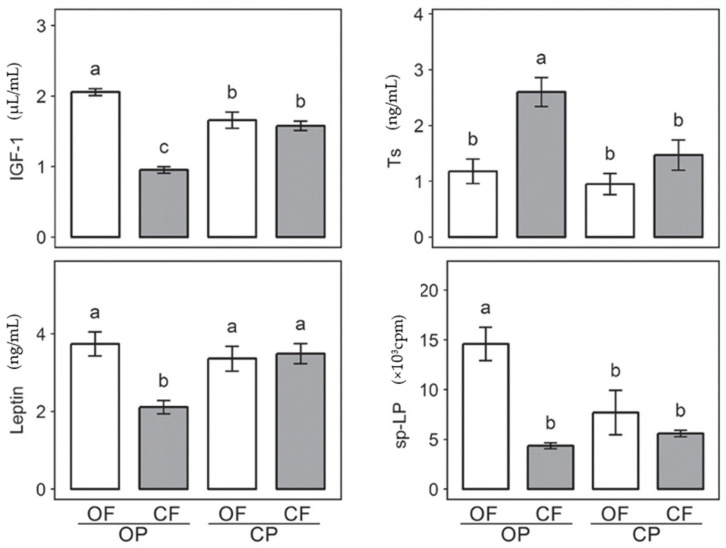
The effects of organic and conventional crop production on four physiological parameters in rats. Plasma concentrations of insulin-like growth factor 1 (IGF-1), testosterone (Ts), leptin and spontaneous lymphocyte proliferation (sp-LP) were measured in 24 Wistar rats after 12 weeks (*n* = 24). Feeds were composed of crops produced from different organic and conventional regimes (OF = organic fertilization, CF = conventional fertilization, OP = organic crop protection, CP = conventional crop protection). Different letters above bar indicate significant difference (*p* < 0.05) as determined by Tukey’s HSD test (a vs. b vs. c). Taken from Baranski et al. (2017) [46], adapted from Srednicka-Tober D et al. (2013) [66].

Another study investigated the effects of organic versus conventional crop fertilization and crop protection schemes on the feed and body composition, hormone balance, and immune activation of rats [66]. Significantly, organic fertilization resulted in a 16% higher white blood cell count, 2.3% higher body protein, and 33% higher plasma glucose compared to mineral fertilization [66]. Further on, feeds produced by organic fertilization increased plasma concentrations of leptin (a hormone involved in regulating energy balance) and insulin-like growth factor (IGF-1, a hormone involved in regulating cell growth and development) by 29% and 46%, respectively, but only when crops were grown under organic crop protection regimes [66]. In contrast, testosterone (Ts) concentrations (a male reproductive hormone) dropped by 45% [66]. Finally, immune reactivity tests demonstrated that spontaneous lymphocyte proliferation increased by 121% for organically fed rats (considering both organic fertilization and crop protection), whereas mitogen-induced lymphocyte proliferation decreased by 47% using organic fertilization; however, this decrease was only observed if crops were grown under conventional crop protection regimes [66]. These results—represented in Figure 4 [46,66]—demonstrate that agronomic practices can significantly influence hormonal and immune parameters in rats, which may in return have profound impacts on the reproductive, metabolic and immune systems of the body. However, it is important to note that the effects of potential confounding factors, such as differences in metabolite bioavailability, were not considered in this study [66]. Overall, these results indicate that high consumption of organic foods may modulate blood nutritional status, perhaps through the increased levels of carotenoids, polyphenols, antioxidants, beneficial fatty acids and other compounds in organic crops that can help regulate important metabolic and immune processes for better human health. Further dietary intervention and prospective cohort studies must be conducted to conclude that these differences in blood nutrient composition have a measurable health benefit to the organic consumer. 

### 4.3. Health Effects Associated with Pesticides

Pesticides can interfere with several molecular pathways through various epigenetic modifications to disturb metabolic and oxidative homeostasis, activate inflammatory pathways, disrupt mitochondrial and endocrine function and dysregulate apoptosis and DNA repair [67]. For individuals exposed to significantly high pesticide concentrations, these molecular changes may aggregate and ultimately lead to an increased risk of obesity, metabolic diseases, cancers, and other chronic diseases. For example, organochlorine pesticides were widely banned following the elucidation of their etiological role in type 2 diabetes [67]. Thus, the health effects of currently authorized pesticides—including organophosphorus, pyrethroids and neonicotinoids—should be thoroughly investigated to inform guidelines on appropriate and responsible pesticide usage. Furthermore, it is important to assess whether the organic diet can reduce exposure to these pesticides and whether this reduced exposure has any benefit to human health. In this section, evidence is presented to highlight the impact of pesticide exposure on different aspects of human health, including fertility, birth outcomes and the incidence of disease. 

### 4.4. Pregnancy-Related Health Characteristics

Nutrition during pregnancy plays a pivotal role in maternal and fetal health, as environmental contaminants in the maternal diet could affect the risk of birth defects through placental or hormonal disturbances. Simões-Wüst et al. [68] assessed the association between organic food consumption and pre-pregnancy health characteristics, revealing that mothers who consumed organic food experienced better health outcomes. These outcomes included a lower risk of overweight and obesity, a more favorable BMI before pregnancy and a lower prevalence of pregnancy-associated diabetes [68]. Furthermore, participants who consumed organic food demonstrated a lower incidence of hypertension compared to non-organic consumers, although the association with blood pressure did not appear to be linear. Notably, blood lipid analysis revealed significantly higher levels of LDL among organic consumers [68].

In a separate study, male newborns of female organic consumers were compared to those of female non-organic consumers regarding hypospadias and cryptorchidism outcomes [69]. While no meaningful association was found between cryptorchidism and organic consumption, there was a lower prevalence of hypospadias among newborns whose mothers consumed organic foods during pregnancy [69]. It is important to highlight that the study classified “organic consumers” as individuals who indicated they sometimes, often, or mostly consumed organic foods in specific categories (vegetables, fruit, bread/cereal, milk/dairy products, eggs, and meat) [69]. For women undergoing infertility treatments, the consumption of fruits and vegetables with high pesticide residues has been associated with lower success rates in achieving clinical pregnancy [70]. Chiu et al. [70] discovered that women consuming more than 2.3 servings per day of such foods had 18% and 26% lower chances of achieving clinical pregnancy and live birth, respectively. This was not significant amongst women who consumed fruits and vegetables with low pesticide residues [70].

Moreover, the reduced exposure to pesticide chemicals through the consumption of organic foods offers additional maternal and fetal health benefits. A study on the consumption frequency of organic vegetables in mid-pregnancy among Norwegian mothers demonstrated that higher consumption of organic foods is associated with a reduced chance of developing preeclampsia [71]. Preeclampsia is present among 5–8% of pregnant women and poses risks of maternal and fetal mortality, an exaggerated inflammatory immune response, and pregnancy-associated hypertension [72]. The study suggests three potential explanations for how organic food consumption reduces preeclampsia risk: decreased exposure to OP pesticides, particularly Chlorpyrifos (CPF), which can increase the permeability of gut intestinal cells to induce inflammation; ingestion of plant secondary metabolites with anti-inflammatory properties, including salicylic acid and polyphenols; and improved intestinal microbiota, resulting in an anti-inflammatory response [71]. 

Overall, there have been several studies that have demonstrated benefits to organic foods either in relation to consumption or the lack of exposure to pesticide chemicals. However, all of these health benefits can only be associated with, but not explained by, an increase in organic food consumption, as differences between study populations and other confounding factors may have influenced the observed results. Therefore, further research is necessary to provide a more comprehensive understanding and draw conclusive evidence regarding measurable health benefits from consuming organic foods during pregnancy. 

### 4.5. Impact on Children’s Health

One of the main draws of the organic diet is that it claims to limit pesticide exposure, which is associated with damaging genotoxic effects including cancer-causing carcinogens and disruptions in the endocrine and nervous systems of the body [73,74]. The toxic effects of pesticide exposure impact fetuses and young children at key developmental stages in their life, leading to life-long effects [36,73,74,75,76]. Further on, OPs and carbamates inhibit acetylcholine breakdown—which is already decreased during pregnancy—and younger children exhibit lower levels of detoxifying enzymes compared to adults, suggesting that young children are especially susceptible to the toxic effects of pesticide exposures [36].

Indeed, the cluster-randomized crossover trial conducted by Makris et al. in 2019 demonstrated that pyrethroid and neonicotinoid pesticide metabolite concentrations were significantly lower in Cypriot children following a 40-day organic diet [73]. Importantly, this outcome was linked to a reduction in various biomarkers of oxidative stress and inflammation [73], suggesting a potential mechanism by which organic foods could confer health benefits to the consumer. 

Similarly, a cross-sectional analysis of data from the National Health and Nutrition Examination Survey (2000–2004) analyzed how dietary exposure to pesticide residues affected ADHD prevalence in U.S. children [36]. The study discovered that a 10-fold increase in urinary concentrations of dimethyl alkylphosphates (OP metabolites) increased the odds of ADHD diagnosis by 55% [36], supporting the theory that OP exposure may influence neurological outcomes at levels common in U.S. children. Conversely, a large prospective birth cohort study of Mexican American children found no association between pesticide exposure and ADHD prevalence [75]. The study assessed the relationship between DAP exposure during utero and mental development index (MDI) scores at 6 months, 12 months and 24 months of age [75]. At 24 months, the authors found that high DAP concentrations during pregnancy were associated with significantly lower MDI score. Interestingly, this study also reported a positive association between postnatal DAP concentrations and MDI index, which should be further explored; however, the chances of pervasive developmental disorder (PDD) were also increased by 2-fold for every 10-fold increase in postnatal DAP concentration [75], suggesting that mental development in children may be impaired in different ways after high prenatal and postnatal exposure to OP metabolites. Other studies [36,73] examining the effects of dietary pesticide exposure have also found similar results, and seemingly agree that following an organic diet protects against elevated pesticide metabolite concentrations in the body.

Considering prenatal exposure to pesticide residues was linked to poorer neurological and cognitive outcomes in children [76], eating organic may play a neuroprotective role and lead to better developmental outcomes. While other studies have criticized that this claim remains unsubstantiated due to the limitations of measuring past exposures and confounding factors such as differences between growing conditions and lifestyle factors, the benefits of the organic diet seem to be reflected in positive health outcomes of study participants and is a promising avenue of research. However, it is important to also consider the potential consequences of recommending an organic diet to children. For example, the higher associated costs of organic fruits and vegetables may discourage the purchasing and consumption of these nutrient-packed foods, which are essential to proper child nutrition and protective against a variety of diseases, including obesity, cardiovascular diseases and cancers [22]. Thus, larger prospective cohort studies should be conducted to draw conclusions about the temporal relationship between dietary pesticide exposure from conventional produce and any toxicity-related effects, and these effects must be weighed against the overall impacts of switching to an organic diet in order to establish a direct health benefit to children.

### 4.6. Risk of Cancers

In a 9.3-year follow-up study [77], the association of organic consumption frequency and cancer incidence was assessed among 623,080 middle-aged women in the United Kingdom. Although previous studies have shown a lower risk of breast and soft tissue cancer among organic consumers, this prospective study revealed no such relationship. The lack of statistical significance could have been affected by potential confounding factors such as lifestyle choices, genetic predispositions or environmental exposures that were not considered in the study. However, there was some evidence that demonstrated that the risk of NHL was reduced by 21% in women who reported usually or always consuming organic food [77].

Another study [78] was designed to assess the overall change in cancer incidence and consumption frequency of organic foods. Following a cohort of 68,946 participants over a mean of 4.6 years, this study revealed that those who consumed organic foods showed a lower risk of NHL (21%, which was similar to the result of a previous study among UK women [77]) and lower risk of postmenopausal breast cancer among participants who consumed organic food frequently (in contrast with the UK study which found no reduction in breast cancer risk) [77,78]. According to this paper, the negative association between organic food consumption and cancer risk was possibly due to lower exposure to synthetic pesticides in organic farming. Specifically, exposure to certain chemicals, such as malathion, terbufos and diazinon has been associated with a 22% higher risk for NHL [78]. The same reasoning can be used to explain the reduced risk of breast cancer; lower exposure to synthetic chemicals may lead to a lower risk for breast cancer among frequent organic food consumers [78].

Exposure to chemical pesticides is also associated with an increased risk of different types of cancers. In the south of Spain, a study [79] on the population of 10 districts, which were categorized based on the potential environmental exposure to pesticides, showed an increased rate of stomach, colorectal, liver, skin, bladder and brain cancer for regions with a higher level of pesticide exposure. In addition, there was an increased rate of prostate, testicular, and lung cancer among male residents in areas where the level of pesticide exposure was high [79]. Many experiments were conducted on the potential carcinogenicity of pesticides using animal models, and these studies have confirmed that the potency of the pesticides and the level of exposure should be considered as factors that increase the risk of cancer development [80]. In animal studies, the carcinogenic potential of some pesticides such as organochlorines, creosote and sulfallate has been observed. Notably, arsenic compounds and insecticides are considered as human carcinogens by the International Agency for Research on Cancer [79]. Together, these studies suggest that exposure to pesticide chemicals, which are extensively used in conventionally grown products, potentiates cancer risk. Thus, eating more organic foods could help reduce exposure to these pesticides and, consequently, potentially also reduce the risk of dangerous human diseases, although the exact link between disease incidence and reduced pesticide exposure is not well established.

Further on, a 2018 Agricultural Health Study (AHS), which assessed the health outcomes of licensed pesticide applicators in North Carolina and Iowa, evaluated the effect of glyphosate on the development of tumors [37]. In their study, 82.8% of 54,251 applicators used glyphosate, but there was no statistically significant link between glyphosate and tumor growth [37]. In spite of this, they found that the highest exposure quartile had an increased risk of acute myeloid leukemia (AML), but this result was not statistically significant [37]. A 2019 meta-analysis of this AHS data and five new case-control studies reported a 41% increased meta-relative risk of NHL for the highest GBH exposure groups [81]. However, a recent review of epidemiological studies published in 2020 criticized the weaknesses of this finding, stating that study discrepancies between exposure groups, the lack of direct comparison between each exposure group, and other epidemiological limitations skew the validity of this data [82]. Thus, the evidence supporting the link between cancer pathogenesis and pesticide exposure is still weak, and further studies are needed to investigate the underlying mechanisms behind these observed associations. 

## 5. Concluding Remarks

Evidence in the current literature suggests that the consumption of organic foods confers promising health advantages for various consumer groups. Multiple statistical analyses have uncovered that organic foods contain significantly higher levels of certain nutrients, including vitamin C, iron and magnesium. Organic food consumption has also shown positive associations with reduced BMI and improved blood nutritional composition across different demographic groups, but these improvements have not been directly linked to specific health outcomes. Further on, organic food has been increasingly popular amongst women due to the claim that they are pesticide-free, and pesticides have been associated with adverse effects on reproductive and immune health. 

While some studies suggest links between pesticide exposure and adverse health effects, conflicting results and methodological limitations challenge our ability to conclusively establish the health benefits of reduced pesticide exposure through organic consumption. The limitations in definitively establishing the health benefits of organic foods stem from various factors including study design flaws, selection bias and other confounding variables. Observational studies comparing organic and non-organic consumers often face challenges such as self-reporting issues, small sample sizes and inconsistent data, hindering the definitive conclusions that can be drawn. Thus, rigorous research, incorporating longitudinal studies and considering diverse influencing factors, is imperative to overcome these limitations and provide a more nuanced understanding of the relationship between organic food consumption and health outcomes. While consumers may consider choosing organic options when convenient, it is premature to recommend organic foods for enhanced health without a more comprehensive understanding of the long-term effects of whole-diet substitutions. Further statistical analyses are necessary to ensure that any recommendations align with robust scientific evidence. Moreover, the call for continued research and policy development is crucial in shaping future nutritional guidelines and regulatory considerations. Continued research, thoughtful policy development and a commitment to rigorous methodologies will contribute to a more informed perspective on the role of organic foods in promoting human health.

## Figures and Tables

**Table 1:** The frequency of glyphosate detection in honey samples from different countries; nd = no data [38].

Country	Number of Samples	Detection Frequency (%)	Minimum (µg/kg)	Mean (µg/kg)	Maximum (µg/kg)
Canada	200	98.5	1	4.9	49.8
Switzerland	16	93.8	<1	4.6	15.9
Estonia	33	12.1	9	35	62
USA	85	28.2	15	92.4	342
Several European Countries	186	12.9	nd	nd	nd

**Table 2:** Comparative analysis of nutrient and salicylic acid content in organic and conventional food products [42,47].

Nutrient	Results	Source
Magnesium, protein, potassium	Assessed mineral element contents in summer corn grain. Organic corn had significantly higher levels of P, Mg and K compared to conventional corn, with increases of 30%, 20% and 30%, respectively (*p* < 0.05). The organic corn showed a higher content of Zn and Fe, although the differences were not statistically significant (*p* > 0.05) Conventional corn grain contained more S and Mn than the organic variety, with levels 15% and 17% higher, respectively	[42]
Salicylic acid is a chemical signal in plants infected by pathogens and is responsible for aspirin’s anti-inflammatory action	The median contents of salicylic acid in the organic and non-organic vegetable soups were 117 (range, 8–1040) ng · g^−1^ and 20 (range, 0–248) ng · g^−1^, respectively The organic soups had a significantly higher content of salicylic acid (*p* = 0.0032, Mann–Whitney U test), with a median difference of 59 ng · g^−1^ (95% confidence interval, 18–117 ng · g^−1^)	[47]

**Table 3:** Comparative analysis of vitamin content in organic and conventional food products [39].

Nutrient	Mean Difference (%)	Significance (*p*-Value)	Number of Studies
Vitamin C	27.0	0.0001	20
Iron	21.1	0.001	16
Magnesium	29.3 (Range: 5–112%)	0.001	17 (Number of Comparisons: 12)
Phosphorus	13.6	0.01	17
Nitrates	15.1	0.0001	18
